# The Soft Coral *Sarcophyton trocheliophorum*: A Warehouse of Terpenoids with Structural and Pharmacological Diversity

**DOI:** 10.3390/md21010030

**Published:** 2022-12-29

**Authors:** Qi-Bin Yang, Qihao Wu, Jun-Kun Chen, Lin-Fu Liang

**Affiliations:** 1 College of Materials Science and Engineering, Central South University of Forestry and Technology, Changsha 410004, China; 2 Department of Chemistry, Yale University, New Haven, CT 06520, USA; 3 Institute of Biomolecular Design & Discovery, Yale University, West Haven, CT 06516, USA

**Keywords:** soft coral, *Sarcophyton trocheliophorum*, terpenoids, chemical structures, pharmacological activities

## Abstract

The soft coral *Sarcophyton trocheliophorum*, which was frequently encountered on Indo-Pacific and Red Sea coral reefs, furnished a wealth of secondary metabolites. Notably, terpenoids dominated the chemical profile of this species. In this review, we summarized the discovery of 156 terpenoids from the soft coral *S. trocheliophorum* specimens in different geographical areas. The structures comprised 13 terpenoidal classes with various functionalities. We covered the era from the first report of *S. trocheliophorum*-derived metabolites in 1976 up to October 2022. The biological effects of these chemical compositions on a vast array of potential pharmacological activities such as protein tyrosine phosphatase 1B (PTP1B) inhibitory, neuroprotective, cytotoxic, anti-inflammatory, antibacterial, antivirus, and immunomodulatory activities were also presented. This review also revealed an immense demand to explore the terpene biosynthetic gene clusters of this species besides the chemo- and bio-investigations.

## 1. Introduction

Taxonomically, the soft coral species *Sarcophyton trocheliophorum* belongs to the genus *Sarcophyton* in the family Alcyoniidae (phylum, Cnidaria; class, Anthozoa; subclass, Octocorallia; order, Alcyonaceae) [1,2]. This benthic soft coral has been frequently encountered on the coral reefs in the Indo-Pacific region, including the South China Sea, Red Sea, and the waters of Okinawa, Indonesia, and Australia [1,2]. Notably, this species has captured the extensive attention of worldwide scientists from China, Australia, India, Saudi Arabia, Egypt, etc., with studies on its chemical composition. Since the first chemical investigation reported in 1976 [3], continuous chemical studies on this soft coral have led to the discovery of numerous secondary metabolites with diverse structural features, implying that *S. trocheliophorum* has become one of the hotspots of marine natural product research. Structurally, these chemical compositions comprise terpenes [4,5,6,7], steroids [8,9,10,11], prostaglandins [12], γ-butenolides [12,13], phenolics [14], etc. Among these metabolites, terpenoids are the dominative constituents. Up to October 2022, 156 terpenoids with various bioactivities have been identified from the soft coral *S. trocheliophorum*.

In order to better understand the medicinal significance of terpenoids from soft coral *S. trocheliophorum*, this review provides the first comprehensive overview of the chemical and/or biological investigations of this soft coral. It covers topics ranging from the distribution of *S. trocheliophorum* to the compound isolation and structural elucidation together with bioactivity evaluation of different types of terpenoids, within a literature survey since its first discovery in 1976 to October 2022.

## 2. Overview of Terpenoids from *S. trocheliophorum*

**Figure 1:**
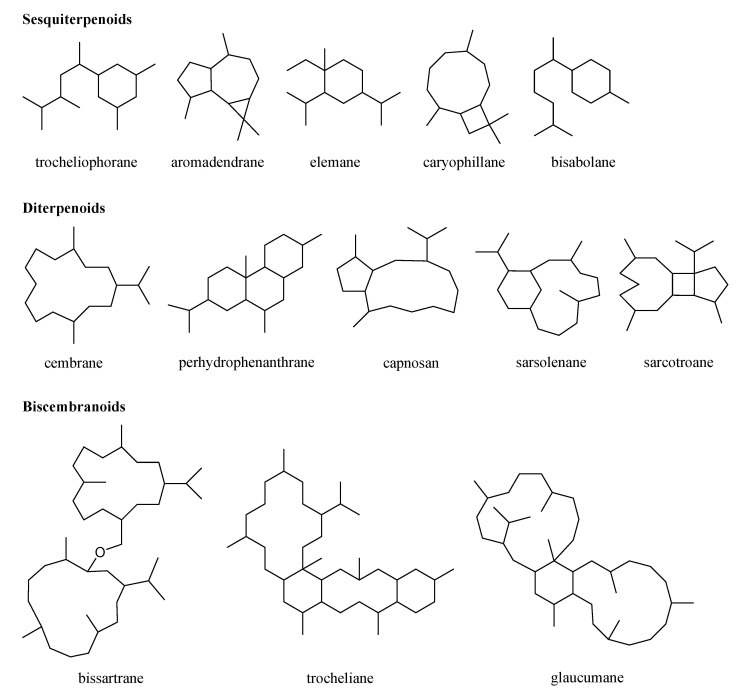
Terpenoidal skeletons from *S. trocheliophorum*.

**Figure 2:**
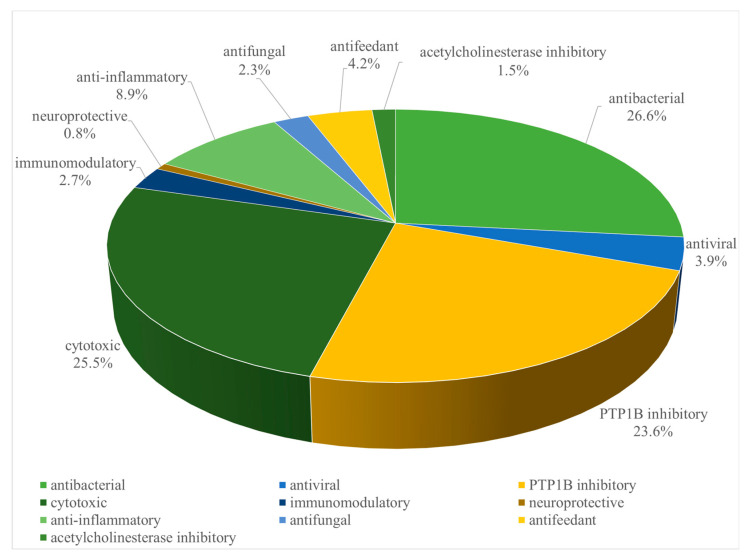
Reported bioactivities of the terpenoids from *S. trocheliophorum*.

As revealed in the literature [3,4,5,6,7,8,9,10,11,12,13,14,15,16,17,18,19,20,21,22,23,24,25,26,27,28,29,30,31,32,33,34,35,36,37,38,39,40,41,42,43,44,45,46,47,48,49,50,51], most of the chemically investigated *S. trocheliophorum* specimens were collected off the islands in the South China Sea and Indian Ocean, and off the coasts of Saudi Arabia, Egypt, and Australia. These collection sites can be divided into three sea waters: the Pacific Ocean, the Indian Ocean, and the Red Sea. Most of the specimens originated from the Pacific Ocean (Table S1) [3,4,5,6,7,8,9,10,11,12,13,14,15,16,17,18,19,20,21,22,23,24,25,26,27,28,29,30,31,32,33,34,35,36,37,38,39,40,41,42,43,44,45,46,47,48,49,50,51]. In this review, terpenoids from *S. trocheliophorum* were classified into three classes: sesquiterpenoids, diterpenoids, and biscembranoids. Among them, diterpenoids were the most common terpenoids from this species, accounting for 89.1% of all descriptions in the literature (Table S1) [3,4,5,6,7,8,9,10,11,12,13,14,15,16,17,18,19,20,21,22,23,24,25,26,27,28,29,30,31,32,33,34,35,36,37,38,39,40,41,42,43,44,45,46,47,48,49,50,51]. These three terpenoid classes were further categorized based on their different skeletal types: trocheliophorane, aromadendrane, elemane, caryophillane, and bisabolane for sesquiterpenoids; cembrane, perhydrophenanthrane, capnosan, sarsolenane, and sarcotroane for diterpenoids; bissartrane, trocheliane, and glaucumane for biscembranoids (Figure 1). Notably, these terpenoids exhibited a broad spectrum of biological activities, such as protein tyrosine phosphatase 1B (PTP1B) inhibitory, neuroprotective, cytotoxic, anti-inflammatory, antibacterial, antivirus, and immunomodulatory activities [3,4,5,6,7,8,9,10,11,12,13,14,15,16,17,18,19,20,21,22,23,24,25,26,27,28,29,30,31,32,33,34,35,36,37,38,39,40,41,42,43,44,45,46,47,48,49,50,51]. As shown in Figure 2, the majority of terpenoids were screened for cytotoxic (including antitumor), antibacterial and PTP1B inhibitory activities [3,4,5,6,7,8,9,10,11,12,13,14,15,16,17,18,19,20,21,22,23,24,25,26,27,28,29,30,31,32,33,34,35,36,37,38,39,40,41,42,43,44,45,46,47,48,49,50,51].

## 3. Sesquiterpenoids

**Figure 3:**
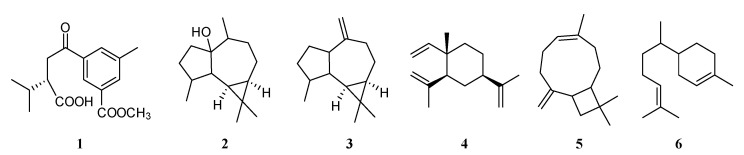
Structures of compounds **1**–**6**, sesquiterpenoids from *S. trocheliophorum*.

Sesquiterpenoids were rarely found in *S. trocheliophorum*. Only six sesquiterpenoids with five different carbon skeletons have been reported (Figure 3). The chemical investigation on the soft coral *S. trocheliophorum* collected from Kurside Island, Indian Ocean, led to the isolation and identification of trocheliophorin (**1**), the first report of sesquiterpenoid from this species [15]. Metabolite **1** was a novel rearranged sesquiterpenoid with an aromatic ring; however, its formation has not been explained on the basis of sequiterpenoid biogenetic considerations. As reported, it could be considered as a degradative product of a diterpenoid. The specimen collected off the Saudi Arabia Red Sea coast at Jeddah afforded an aromadendrane sesquiterpenoid palustrol (**2**) [16]. This tricyclic sesquiterpenoid **2** possessed a broad spectrum of biological activities including antibacterial, antifungal, antifeedant, and antitumor effects. The antimicrobial bioassay revealed that **2** showed moderate activities against *Staphylococcus aureus*, *Staphylococcus epidermidis*, *Staphylococcus aureus* MRSA, *Micrococcus* spp., *Streptococcus pneumonia*, *Acnietobacter* spp., *Klebsiella pneumonia*, *Pseudomonas aeruginosa*, and *Escherichia coli* with diameters of inhibition zones ranging from 8 to 11 mm. Meanwhile, **2** had moderate antifungal activities against *Candida albicans*, *Candida tropicals*, *Aspergillus flavus*, and *Aspergillus niger* (the diameters of inhibition zones were 8–12 mm). Up to 11.1 μM, no toxicity was recorded against *Artimia salina* as a tested organism for **2**. Additionally, **2** showed antitumor activities with LD_50_ of 2.8 and 3.1 μM for the two tested lymphoma and Erlish cell lines. Another related aromadendrane sesquiterpenoid alloaromadendrene (**3**) together with *β*-elemene (**4**), *trans*-caryophyllene (**5**), and bisabolene (**6**) were isolated from the specimen collected off the east Egypt Red Sea coast at Hurgada [17].

## 4. Diterpenoids

**Figure 4:**
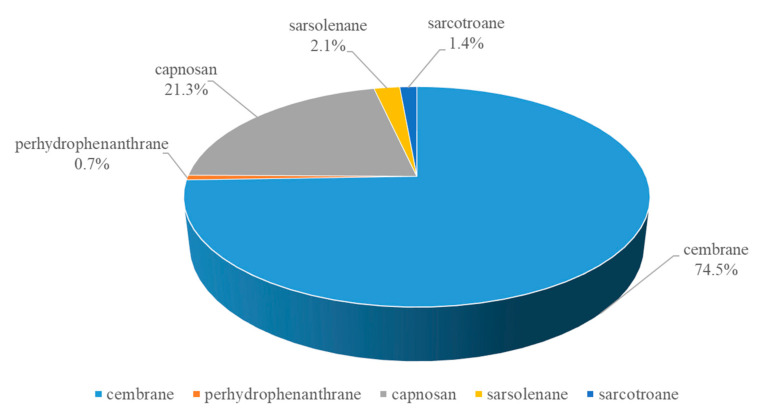
Diterpenoids from *S. trocheliophorum* with different structural types.

Diterpenoids are the most abundant secondary metabolites in *S. trocheliophorum* [3,4,5,6,7,8,9,10,11,12,13,14,15,16,17,18,19,20,21,22,23,24,25,26,27,28,29,30,31,32,33,34,35,36,37,38,39,40,41,42,43,44,45,46,47,48,49,50,51]. They showed a variety of biological activities including antitumor, anti-inflammatory, antibacterial, and antifeedant effects [3,4,5,6,7,8,9,10,11,12,13,14,15,16,17,18,19,20,21,22,23,24,25,26,27,28,29,30,31,32,33,34,35,36,37,38,39,40,41,42,43,44,45,46,47,48,49,50,51]. The broad bioactivities implied that these secondary metabolites might play important ecological functions in corals’ lives such as antifeedant defenses. At present, diterpenoids from *S. trocheliophorum* can be categorized into five carbo-skeleton types: cembrane, perhydrophenanthrane, capnosane, sarsolenane, and sarcotroane [3,4,5,6,7,8,9,10,11,12,13,14,15,16,17,18,19,20,21,22,23,24,25,26,27,28,29,30,31,32,33,34,35,36,37,38,39,40,41,42,43,44,45,46,47,48,49,50,51]. As indicated in Figure 4, the majority of the diterpenoids were cembrane diterpenoids.

### 4.1. Cembrane Diterpenoids

The worldwide investigations of soft coral *S. trocheliophorum* yielded a whole variety of cembranoids with different degrees of oxidation. One or more of the methyl groups were oxidized to a hydroxymethyl group or a carboxyl group. These groups usually appeared as methyl esters, five- to seven-membered oxacycles, and lactone moieties. Herein, this review encompasses the different categories of cembrane diterpenoids as follows: isopropyl/isopropenyl, furane, pyrane, *γ*-lactone, *ε*-lactone, and other miscellaneous.

#### 4.1.1. Isopropyl/Isopropenyl Cembranoids

**Figure 5:**
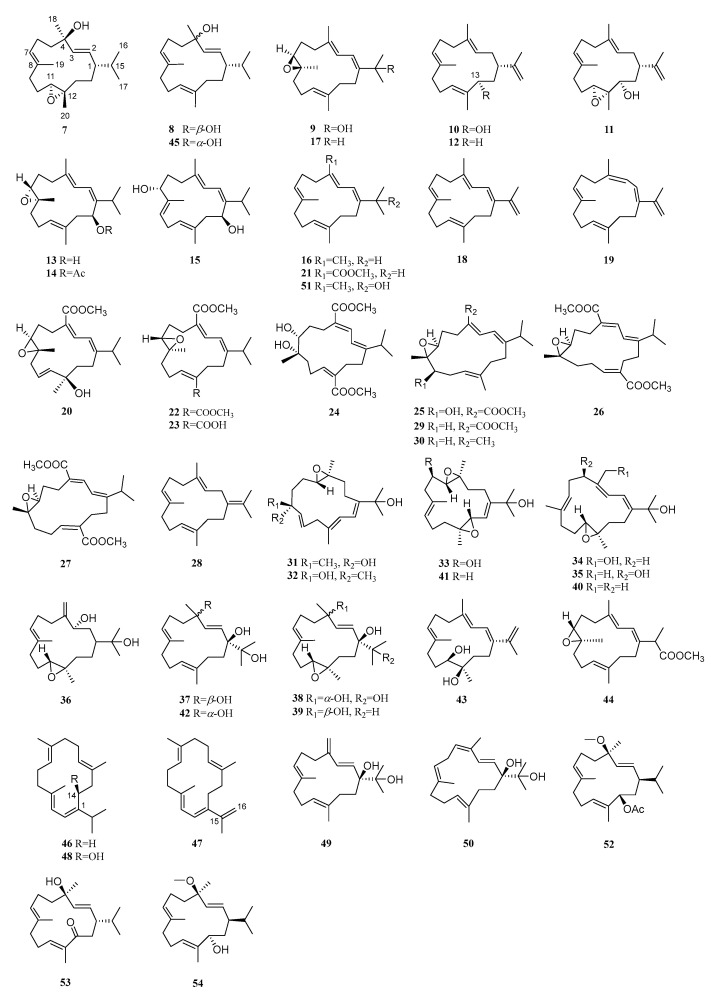
Structures of compounds **7**–**54**, isopropyl/isopropenyl cembranoids from *S. trocheliophorum*.

At present, 48 isopropyl/isopropenyl cembranoids from the soft coral *S. trocheliophorum* distributed in different geographical locations have been disclosed (Figure 5).

In the first chemical study of *S. trocheliophorum* in 1976 by Tursch, an isopropyl cembrane diterpenoid trocheliophorol (**7**) was discovered from the soft coral collected in the Seychelles Islands [3]. At first, only the absolute configuration of C-1 was determined to be 1*S* [18]. Later, the absolute configurations of C-4, C-11, and C-12 were elucidated as 4*R*,11*S*,12*S* by Kashman et al. [19] and Coll et al. [20], respectively. In addition, trocheliophorol (**7**), thunbergol (**8**), and 7,8-epoxy-1*E*,3*E*,11*E*-cembratriene-15-ol (**9**) were found in the sample collected on Orpheus Island [20]. These isolates played a defensive role against the sea slug predator *Phyllodesmium longicirra*.

In 1985, Elyakov and co-workers reported two related isopropenyl cembrane diterpenoids 13*S*-hydroxy-(-)-neocembrene (**10**) and 13*S*-hydroxy-11,12-epoxy-(-)-neocembrene (**11**) from another collection of this soft coral on the reefs of the Seychelle Islands [21]. The X-ray diffraction analysis of the well-crystallized *p*-nitrobenzoate of **8** together with the successful application of Horeau’s method established the 13*S* absolute configuration for **10**. Epoxidation of the alcohol **10** yielded epoxyalcohol **11**, revealing the 11*S*,12*S* absolute configurations for **11**. In Elyakov’s further study on this specimen, the existence of neocembrene (**12**) was confirmed [22]. The cytostatic activities of compounds **10**–**12** were determined using a radiometric method in a micro-modification from the level of inclusion of [^3^H]thymidine, [^14^C]uridine, and [^3^H]leucine in the acid-insoluble fraction of Ehrlich cells. The results showed that **10** and **11** caused 50% inhibition of the incorporation of [^3^H]thymidine, [^14^C]uridine, and [^3^H]leucine at concentrations of 19.5–49.0 μg/mL, of which **10** displayed higher cytostatic activity. Compound **12** exhibited no cytostatic activity at concentrations of up to 50 μg/mL. This result revealed that the hydroxyl group substituted at C-13 exhibited strong impacts on the cytostatic activity based on the comparison of these three isopropenyl cembranoids. The permeability of the liposomes study showed that diterpene **10** was the most effective inductor of the release of [1-^14^C]-glucose from the liposomes.

An investigation of metabolites from the soft coral *S. trocheliophorum*, collected on Orpheus Island, yielded (7*R*,8*R*,14*S*,1*E*,3*E*,11*E*)-7,8-epoxycembra-l,3,11-trien-14-ol (**13**), (7*R*,8*R*,l4*S*,l*E*,3*E*,11*E*)-14-acetoxy-7,8-epoxycembra-l,3,11-triene (**14**), and (7*R*,14*S*,1*E*,3*E*,8*E*,11*E*)-cembra-1,3,8,11-tetraene-7,14-diol (**15**), whose absolute configurations were determined using Mosher’s method [23]. Both **13** and **14** showed similar cytotoxic profiles with IC_50_ values of 2.5 μg/mL against murine leukemia P388, 5 μg/mL against human lung carcinoma A549, 10 μg/mL against human colon carcinoma HT29, and 10 μg/mL against human melanoma MEL28 cell lines. An examination of a Singaporean specimen resulted 7,8-epoxy-1*E*,3*E*,11*E*-cembratriene-15-ol (**9**), isoneocembrene (**16**), and 7,8-epoxy-1*E*,3*E*,11*E*-cembratriene (**17**) [8]. In the bioassay, cembratertraene **16** showed moderate growth inhibitory activity against human HL60 leukemia, M14 skin melanoma, and MCF7 breast carcinoma cells with EC_50_ values of 37.2, 74.6, and 72.8 μg/mL, respectively. Both cembratrienes **9** and **17** exhibited a moderate inhibitory effect on the HL60 cell (EC_50_ = 34.2 and 63.8 μg/mL, respectively), whereas only **9** displayed moderate inhibitory activity against the MCF7 cell with an EC_50_ value of 54.6 μg/mL.

Two new secondary metabolites, yalongenes A (**18**) and B (**19**), were isolated from the South China Sea soft coral *S. trocheliophorum* collected in Yalong Bay, Hainan Island [24]. Compounds **18** and **19** were tested for their cytoprotective effects on SH-SY5Y cell injury induced by hydrogen peroxide, and the results showed that compound **19** had significant cytoprotective activity at the concentration of 1.0 μM. It appeared that the *Z*-configuration of the conjugated olefinic bond Δ^3^ played a key role for the cytoprotective activity of isopropenyl cembranoids due to the observation of considerable activity for **19** and no activity for **18**. From another collection of this soft coral on the reefs of Yalong Bay, sarcophytonolide M (**20**) was found as an undescribed cembranoid [4]. This isolate was inactive in the protein tyrosine phosphatase 1B (PTP1B) bioassay, a key target for the treatment of type-II diabetes and obesity. To search for more cembrane diterpenoids with PTP1B inhibitory activity, a much more detailed investigation of this specimen was conducted, resulting in the isolation of an array of cembranoids including undescribed sarcophytonolides N–Q (**21**–**24**) [25] and S–U (**25**–**27**) [26], together with known analogs cembrene-C (also termed as isoneocembrene, **16**), (*E*,*E*,*E*)-1-isopropenyl-4,8,12-trimethylcyclotetradeca-3,7,11-triene (**28**) [25], sarcophytonolide A (**29**), and (*E*,*E*,*E*)-7,8-epoxy-l-isopropyl-4,8,12-trimethylcyclotetradeca-l,3,11-triene (**30**) [26]. For compounds **25** and **27**, solution time-dependent density functional theory (TDDFT) calculations of ECD and specific rotation were applied in combination with conformational and NMR data analysis to determine their absolute configurations. Compounds **16** and **21**–**28** were tested for their inhibitory activity against PTP1B. The results revealed that sarcophytonolide N (**21**) showed significant activity with the IC_50_ value of 5.95 μM; compound **16** showed moderate activity (IC_50_ = 26.6 μM), while the remaining compounds **22**–**28** were inactive (IC_50_ > 20 μg/mL). None of the tested compounds (**22**–**25**, **28**) exhibited cytotoxicity against two human cell lines A-549 and HL-60. To exploit the minor chemical constituents, research on the other two *S. trocheliophorum* specimens was carried out and yielded sarcophytrols D–L (**31**–**39**) [27,28], 11,12-epoxy-1(*E*),3(*E*),7(*E*)-cembratrien-15-ol (**40**), sinugibberol (**41**) [27], crassumol A (**42**) [28], sarglaucol (**43**) [29], 7R^*^,8R^*^-epoxy-isoneocembyance A-16-oic methyl ester (**44**), and 4-*epi*thunbergol (**45**) [30]. None of the diterpenoids **31**–**43** showed PTP1B inhibitory effects in the bioassay. The cytotoxicity and acetylcholinesterase inhibitory bioassays of compounds **44** and **45** were also evaluated, and only **45** exhibited weak cytotoxicity against A-549 and HL-60 cell lines.

It was interesting to find that the Red Sea soft coral *S. trocheliophorum* produced compound **16** [16]. This cembratetraene was screened for a broad spectrum of biological activities including antibacterial, antifungal, cytotoxic, and antitumor effects. The antimicrobial bioassay revealed that **16** showed moderate activities against *S. aureus* MRSA, *Micrococcus* spp., *S. pneumonia*, and *Acnietobacter* spp. (the diameters of inhibition zones were 8–10 mm). Meanwhile, **16** had moderate antifungal activities against *C. albicans*, *C. tropicals*, *A. flavus,* and *A. niger* with diameters of inhibition zones ranging from 11 to 17 mm. Up to 13.6 μM, no toxicity was recorded against *A. salina* for **16**. Additionally, **16** did not exhibit antitumor activities against the two tested lymphoma and Erlish cell lines. Another Red Sea collection afforded two new non-polar diterpenes, *cis*-cembrene C (**46**) and *cis*-cembrenene C (**47**) [17], and one known analog: (+)-sarcophytol A (**48**) [31]. These isolates were inactive against *Bacillus subtilis*, *S. aureus*, *Streptomyces viridochromogenes* (Tü 57), *E. coli*, *C. albicans*, *Mucor miehi*, *Chlorella vulgaris*, *Chlorella sorokiniana*, *Scenedesmus subspicatus*, *Rhizoctonia solani*, and *Pythium ultimum* at 40 *μ*g/disk. They were further examined for cytotoxicity against brine shrimp at a concentration of 10 μg/mL (24 hr). Only *cis*-cembrene C (**46**) showed weak cytotoxicity with the mortality of 22.5%. This revealed that the hydroxyl group at C-14 and the exomethylene Δ^15^ were not necessary for the antifeedant property.

Recently, two new cembranoids, ximaosarcophytols A (**49**) and B (**50**), along with three related known ones, sarcophytrol J (**37**), crassumol A (**42**), and 15-hydroxycembra-1,3,7,11-tetraene (**51**), were found in the soft coral *S. trocheliophorum* collected from Ximao Island, South China Sea [32]. The absolute configurations of these two new compounds were elucidated using TDDFT/ECD calculations. All of the metabolites were subjected to various bioassays such as cytotoxic, inhibitory effects against demethylases ALKBH3 and FTO/ALKBH5, etc. However, all tested compounds showed only moderate to weak activities. The investigation of the specimen collected from the coast of the Xisha Islands in the South China Sea yielded 1,13-di-*epi*-13-acetoxy launine P (**52**), 13-oxo-thunbergol (**53**), and launine P (**54**) [33]. ECD calculations were also performed to assign the absolute configurations for new compounds **52** and **53**. All of the isolates showed weak antibacterial activity against five pathogenic bacteria, *S. aureus* CMCC (B) 26003, *methicillin-resistant S. aureus* (MRSA) ATCC43300, *B. subtilis* CMCC (B) 63501, *P. aeruginosa* CMCC (B) 10104, and *Salmonella paratyphi* CMCC (B) 50071. Furthermore, all of these secondary metabolites were tested in vitro for antiviral activity against influenza A virus H1N1. Moderate anti-H1N1-virus activity was observed for terpenoids **53** and **54** (96.2% and 89.4% inhibition at 30 μM, respectively).

#### 4.1.2. Furane Cembranoids

**Figure 6:**
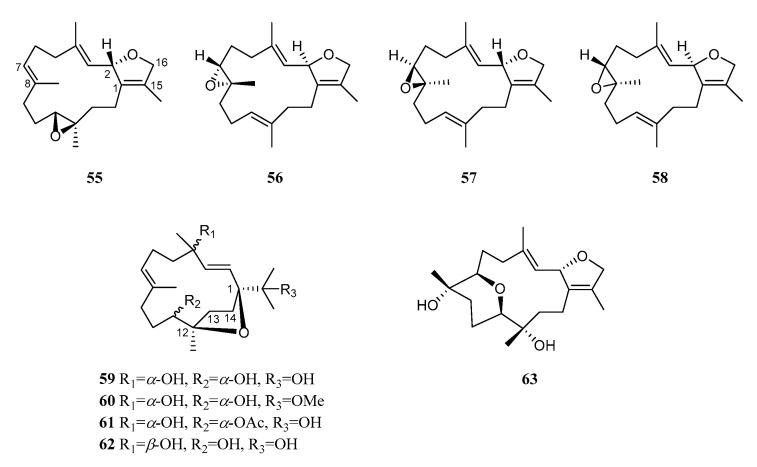
Structures of compounds **55**–**63**, furane cembranoids from *S. trocheliophorum*.

Records of furane cembranoids from *S. trocheliophorum* are rare, with only nine reported so far (Figure 6). According to the location of furan rings, they can be divided into two categories. Type I furane cembranoids possess an ether bridge between C-2 and C-16 while type II furane cembranoids have an ether bridge between C-1 and C-12.

In the first report of the chemical investigation of this species, sarcophytoxide (**55**) and isosarcophytoxide (**56**) were found in the Indonesian collection [3]. Compound **55** was also present in the Australian specimen collected from Orpheus Island [23], while **56** was disclosed in a Formosan sample collected in Kenting [34]. Compound **56** exhibited significant cytotoxicity against A-549, HT-29, KB, P-388, and HL-60 cells with ED_50_ values of 8.23, 8.27, 9.98, 0.49, and 0.80 μg/mL, respectively. A further study on a Formosan collection led to a cembranoid: 16-deoxysarcophine (**57**) [35]. Compound **57** showed potent cytotoxicity against A549, HT-29, and P-388 cells (ED_50_ = 15.74, 16.07, 3.87 μg/mL, respectively), but was inactive against the KB cell (ED_50_ > 50 μg/mL). A preliminary analysis of the structure–activity relationships revealed that the *S* configuration of H-2 decreased the cytotoxicity against the above-mentioned cells based on the comparison of furane cembranoids **56** and **57**.

An investigation of the soft coral *S. trocheliophorum* collected off the cost of Hainan Island in the South China Sea resulted in (–)-sarcophytoxide (**58**) [30]. Compound **58** was inactive in the cytotoxicity and acetylcholinesterase inhibitory bioassays. An examination of another South China Sea specimen yielded sarcophytrols M–P (**59**–**62**) [29]. The modified Mosher method was applied to determine the absolute configuration of sarcophytrol M (**59**). Unfortunately, the bioassay results indicated that these metabolites exhibited no obvious bioactivities (PTP1B inhibitory activity, cytotoxicity against the human tumor cell lines HL-60 and K-562, antibacterial activity against *P. aeruginosa*).

Recently, a new cembrane diterpene, isocrassumol B (**63**), was encountered in a South China Sea sample collected on the Xisha Islands [33]. A pyran motif, rarely occurring between C-7 and C-11, was embodied in the structure of **63**, in addition to the furan ring formed through C-2 and C-16. Its absolute configuration 2*S*,7*R*,8*S*,11*R*,12*S* was assigned using the ECD calculation. An antibacterial evaluation of **63** showed no activity against five pathogenic bacteria, *S. aureus* CMCC (B) 26003, *methicillin-resistant S. aureus* (MRSA) ATCC43300, *B. subtilis* CMCC (B) 63501, *P. aeruginosa* CMCC (B) 10104, and *S. paratyphi* CMCC (B) 50071. In addition, **63** did not exhibit in vitro antiviral activity against influenza A virus H1N1.

#### 4.1.3. Pyrane Cembranoids

**Figure 7:**
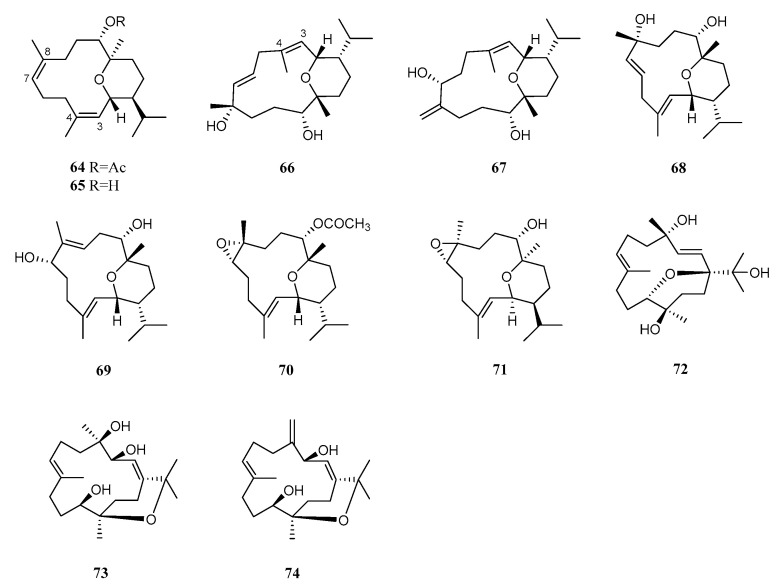
Structures of compounds **64**–**74**, pyrane cembranoids from *S. trocheliophorum*.

Pyrane-based cembranoids are also rare with only eleven members discovered up until now (Figure 7). Based on the different locations of the pyran rings, these metabolites can be categorized into three classes as follows: (type I) cyclized across C–2 and C-12; (type II) across C–1 and C-11; (type III) across C–12 and C-15.

Four new rare pyrane-based cembranoids, sarcotrocheliol acetate (**64**), and sarcotrocheliol (**65**) were isolated from the Red Sea soft coral *S. trocheliophorum* collected off the cost of Jeddah, Saudi Arabia [16]. It might be worth pointing out that the absolute configuration of sarcotrocheliol (**65**) was determined on the basis of a single crystal X-ray analysis in another investigation [36]. This result established the configuration revision of two chiral centers C-5 and C-14 as 5*S*,14*R*, which were misassigned in a previous report [16]. These two cembranoids were evaluated for a broad spectrum of biological activities including antibacterial, antifungal, antifeedant, and antitumor effects. The antimicrobial bioassay revealed that both compounds exhibited strong activities against *S. aureus*, *S. epidermidis*, *S. aureus* MRSA, *Micrococcus* spp., *S. pneumonia*, *Acnietobacter* spp., *K. pneumonia*, *P. aeruginosa*, and *E. coli* with diameters of inhibition zones ranging from 12 to 18 mm. However, none of them showed toxicity against *A. salina* or antitumor activity against lymphoma and Erlish cell lines up to 15.3 μM [16]. Continuous study on this specimen led to two additional new cembrane diterpenoids: sarcotrocheldiols A (**66**) and B (**67**) [37]. Only weak antibacterial activity was recorded for compounds **66** and **67** against the tested pathogenic bacteria including *Acinetobacter baumannii*, *E. coli*, *K. pneumonia*, *P. aeruginosa*, *S. aureus*, *S. epidermidis*, and *S. pneumoniae*. It seemed the *E*-geometry of Δ^7^ of pyrane cembranoids could increase the antibacterial activity.

In addition to three new cembranoids 9-hydroxy-10,11-dehydro-sarcotrocheliol (**68**) [31], 9-hydroxy-7,8-dehydro-sarcotrocheliol (**69**), and 8,9-expoy-sarcotrocheliol acetate (**70**) [38], sarcotrocheliol acetate (**64**) was also present in another Red Sea soft coral sample collected off the coast of Hurghada, Egypt. It is noteworthy that the absolute configurations of **69** were determined as 1*S*,4*R*,5*S*,9*R*,14*R* using single crystal X-ray analysis in another study [36]. This study revealed a reverse configuration at the three chiral centers C-1, C-4, and C-9, which was misassigned in a previous report [38]. All of the reported compounds showed no activity in the antimicrobial activity testing against *S. aureus, B. subtilis* ATCC6051, *S. viridochromogenes* Tü 57, *E. coli, Mucor miehei* Tü 284, *C. albicans,* and the green alga *C. vulgaris* at the concentration of 40 μg/disc [31,38]. The recollection of this soft coral off the Egyptian Red Sea coast afforded sarcotrocheliol (**65**) together with an unreported sarcopyranoid A (**71**) [39]. Compounds **65** and **71** displayed low cytotoxicity against A549 (IC_50_ 67.5 ± 3.9, 79.9 ± 2.8 μg/mL, respectively). It appeared that the replacement of the olefinic bond at C-7/C-8 by an epoxide functionality did not cause the loss of the cytotoxicity for these two pyrane cembranoids. However, none of them exhibited detectable antileishmanial activity (IC_50_ > 100 μg/mL).

An examination of the South China Sea soft coral *S. trocheliophorum* disclosed three new bicyclic cembranoid sarcophytrols Q–S (**72**–**74**) [29]. Among them, sarcophytrols R (**73**) and S (**74**) shared a rare bicyclic skeleton of the decaryiol type. The bioassay results showed that none of them displayed PTP1B inhibitory activity or cytotoxicity against the human tumor cell lines HL-60 and K-562 or antibacterial activity against *P. aeruginosa.*

#### 4.1.4. *γ*-Lactone Cembranoids

**Figure 8:**
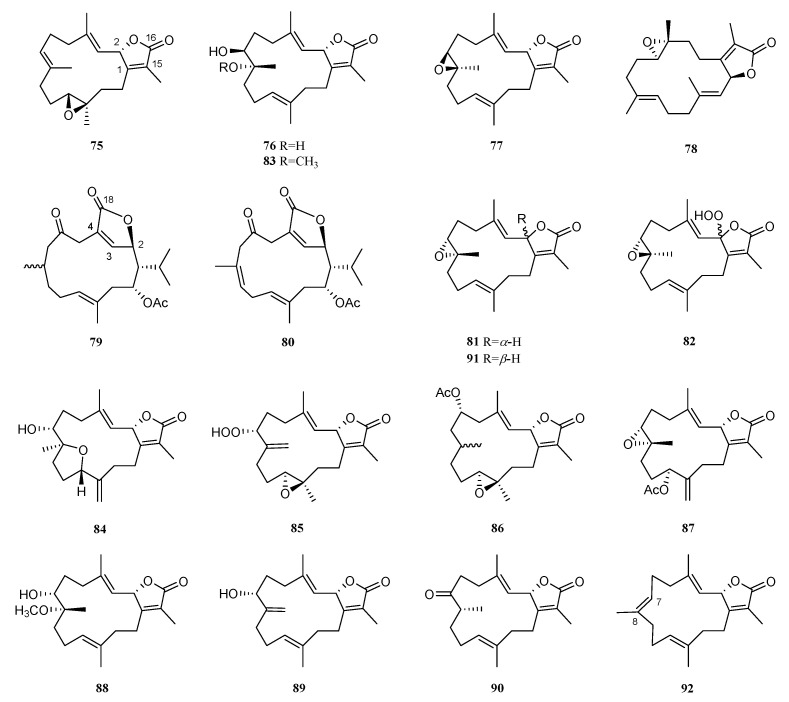
Structures of compounds **75**–**92**, *γ*-lactone cembranoids from *S. trocheliophorum*.

These metabolites can be regarded as the oxidation products of furane-based cembranoids. So far, fifteen diterpenoids of this type have been reported in *S. trocheliophorum* (Figure 8). According to the location of the *γ*-lactone ring, these cembranoids can be divided into two classes: ester linkage via C-2 and C-16 (type I) and via C-2 and C-18 (type II).

A cytotoxicity-guided fractionation of Formosan soft coral *S. trocheliophorum* yielded (+)-isosarcophine (**75**) [34]. This metabolite displayed potent cytotoxicity against A-549, HT-29, KB, P-388, and HL-60 cells with ED_50_ values of 13.32, 16.88, 24.54, 0.73, and 6.73 μg/mL, respectively. A further study on a Formosan sample led to the isolation of two cembranoids: 7*β*,8*α*-dihydroxydeepoxysarcophine (**76**) and sarcophine (**77**) [35]. Compounds **76** and **77** showed significant cytotoxicity against A549, HT-29, KB, and P-388 cells with ED_50_ values ranging from 2.42 to 24.66 μg/mL. It was interesting to find that the opening of the epoxide ring did not cause the loss of cytotoxicity against these cells for these two *γ*-lactone cembranoids. However, they were inactive against the KB cell (ED_50_ > 50 μg/mL), indicating their possible selective cytotoxicity. Interestingly, sarcophine (**77**) was distributed in many samples of different inhabiting environments such as Singaporean [8] and Saudi Arabian [16] waters. Compound **77** exhibited moderate growth inhibitory activity against the human HL60 leukemia cell (ED_50_ = 61.8 μg/mL) in the study on the Singaporean specimen [8]. Metabolite **77** was tested for antibacterial, antifungal, antifeedant, and antitumor effects in the research on a Saudi Arabian sample. The antimicrobial bioassay revealed that **77** showed moderate activities against *S. aureus*, *S. epidermidis*, *S. aureus* MRSA, *Micrococcus* spp., *S. pneumonia*, *Acnietobacter* spp., *K. pneumonia*, and *E. coli* with diameters of inhibition zones ranging from 8 to 12 mm. Meanwhile, **77** had moderate antifungal activities against *C. albicans*, *C. tropicals*, *A. flavus,* and *A. niger* (the diameters of inhibition zones were around 10 mm). Up to 9.4 μM, no toxicity was recorded against *A. salina* for the natural product **77**. Additionally, **77** showed antitumor activities with LD_50_ values of 2.5 and 3.8 μM for the two tested lymphoma and Erlish cell lines, respectively [16].

An investigation of South China Sea soft coral *S. trocheliophorum* disclosed an unreported cembranoid (-)-sartrochine (**78**) [40]. Its relative stereochemistry was determined using X-ray diffraction analysis, and its absolute configuration was assigned using Cotton effects analysis. It displayed cytotoxic activity against S_180_ cells (IC_50_ = 12.3 μg/mL) and an antibiotic effect on *Streptococcus hemolyticus* (MIC = 14 μg/mL). Sarcophytonolides J (**79**) and R (**80**) were reported from another South China Sea sample [25]. The locations of *γ*-lactone on C-2, C-3, C-4, and C-18 were rare in cembranoids. In the PTP1B inhibitory bioassay, compounds **79** and **80** were inactive (IC_50_ ≥ 20 μg/mL). Additionally, none of them exhibited cytotoxicity against two human cell lines, A-549 and HL-60. Continuous study on the third South China Sea specimen led to the discovery of *ent*-sarcophine (**81**) and 2-hydroperoxysarcophine (**82**) [30]. The cytotoxicity against A-549 and HL-60 cell lines and acetylcholinesterase inhibitory bioassays of compounds **81** and **82** were evaluated, while only **81** exhibited weak acetylcholinesterase inhibitory activity. This result revealed that the hydroperoxide at C-2 might decrease the acetylcholinesterase inhibitory activity for *γ*-lactone cembranoids.

A new cembranoid, trocheliol (**83**), was isolated from the cultured soft coral *S. trocheliophorum*, which was originally collected off the coast of Pingtung, a southern Taiwan island [41,42]. It was noted that **83** was the first example of an *α*,*β*-unsaturated *γ*-lactone cembranoid possessing a tetrahydrofuran moiety with a rare 8,11-ether linkage. A hydroperoxycembranoidal diterpene trocheliolide A (**84**) [43] and its analog trocheliolide B (**85**) [44] were obtained from another Formosan soft coral *S. trocheliophorum*, which was collected off the cost of Lanyu Island. The cytotoxicity of trocheliolide A (**84**) against the proliferation of a limited panel of cancer cell lines, including MOLT-4 (human acute lymphoblastic leukemia), SUP-T1 (human T-cell lymphoblastic lymphoma), DLD-1 (human colorectal adenocarcinoma), LNCaP (human prostatic carcinoma), and MCF7 (human breast adenocarcinoma) was studied. The results showed that **84** was not cytotoxic against the above cancer cells (IC_50_ > 20 μg/mL).

An examination of the Egyptian specimen from the Gulf of Suez led to the discovery of a methyl ether derivative of **76**, 7*β*-hydroxy-8*α*-methoxydeepoxysarcophine (**86**) [45]. A chemical investigation of another Egyptian sample from the coast of Hurghada resulted in the isolation of trochelioids A (**87**), B (**88**), 16-oxosarcophytonin E (**89**), and 8-*epi*-sarcophinone (**90**), together with two which were previously reported: sarcophine (**77**) and *ent*-sarcophine (**81**) [46]. Recently, a new cembranoid 7*α*,8*α*-sarcophine (**91**) and the known sarcophytonin B (**92**) were found in this species collected off the cost of the Xisha Islands, South China Sea [33]. ECD calculations were performed to determine the configuration of 7*α*,8*α*-sarcophine (**91**) as 2*S*,7*R*,8*S*. The antibacterial evaluations showed that only sarcophytonin B (**92**) exhibited a strong inhibition against *S. aureus* and *B. subtilis* (MIC < 0.5 μg/mL). The olefinic bond Δ^7^ of the *γ*-lactone cembranoid might play a crucial role in the antibacterial activity against the selected bacteria considering the decreased activity of compound **91** compared to that of **92**. Unfortunately, both were inactive against influenza A virus H1N1.

#### 4.1.5. *ε*-Lactone Cembranoids

**Figure 9:**
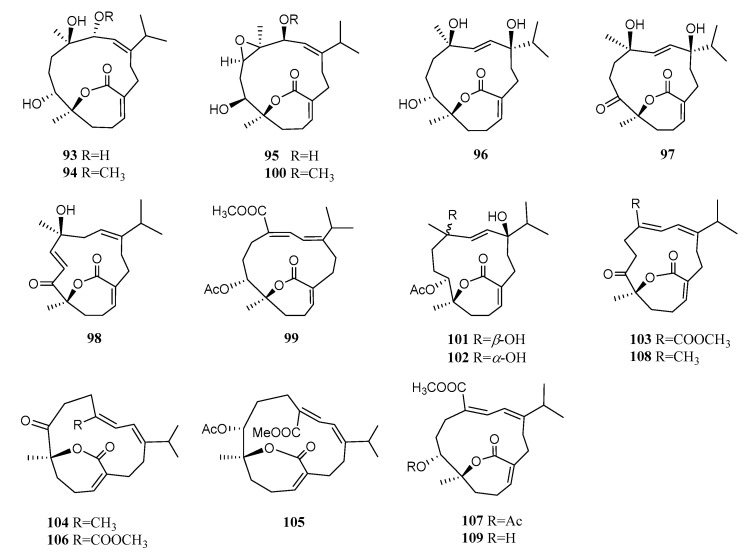
Structures of compounds **93**–**109**, *ε*-lactone cembranoids from *S. trocheliophorum*.

Sixteen cembranoids possessing an *α*,*β*-unsaturated *ε*-lactone have been reported (Figure 9). These metabolites including eleven new compound sartrolides A–J (**93–102**) [26,47], together with seven known analogs, ketoemblide (**103**), 4*Z*,12*Z*,14*E*-sarcophytolide (**104**), sarcrassins D (**105**) and E (**106**), emblide (**107**), sarcophytolide (**108**), and deacetylemblide (**109**) [25,26,47], were obtained from the samples collected from the South China Sea. A solution TDDFT calculation of ECD was applied to determine the absolute configuration of **100** [26]. The absolute configurations of **104**, **105,** and **107** were assigned using the solid-state TDDFT-ECD approach and a single crystal X-ray diffraction experiment with Cu K*α* radiation [26,47]. It is noteworthy that these diterpenoids shared the same *R* configuration at C-8, which was the joining point of the *α*,*β*-unsaturated *ε*-lactone moiety on the cembrane ring. Compounds **100**, **103,** and **104** showed potent PTP1B inhibitory activity (IC_50_ = 19.9, 27.2, 15.4 μM, respectively). The methyl ester of *ε*-lactone cembranoids might play an important role in the PTP1B inhibitory activity considering the increased activity of compound **100** compared to that of **95**. Moreover, compounds **104** and **108** exhibited antibacterial activity against the methicillin-sensitive *Staphylococcus aureus* Newman strain (MIC = 250, 398 μM, respectively). A preliminary analysis of structure–activity relationships revealed that the ester group dramatically decreased the antibacterial activity for *ε*-lactone cembranoids based on the comparison of two pairs of **103**/**108** and **104**/**106**. Unfortunately, none of them displayed cytotoxicity against two human A-549 and HL-60 cell lines.

#### 4.1.6. Other Miscellaneous Cembranoids

**Figure 10:**
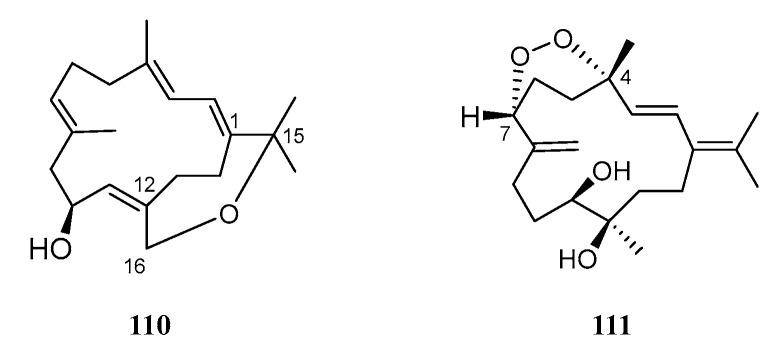
Structures of compounds **110** and **111**, other miscellaneous cembranoids from *S. trocheliophorum*.

Two new bicyclic cembranoids, sarcophytrols T (**110**) and U (**111**), were isolated from the South China Sea soft coral *S. trocheliophorum* [29] (Figure 10). Their chemical structures only differed at the patterns of the oxacycle (oxepane for **110** and peroxyl ring for **111**). The absolute configuration of **110** was determined using the modified Mosher method. The PTP1B inhibitory, antitumor, and antibacterial activities were evaluated for these two diterpenoids. Unfortunately, none of them were active in the bioassays.

### 4.2. Perhydrophenanthrane Diterpenoid

**Figure 11:**
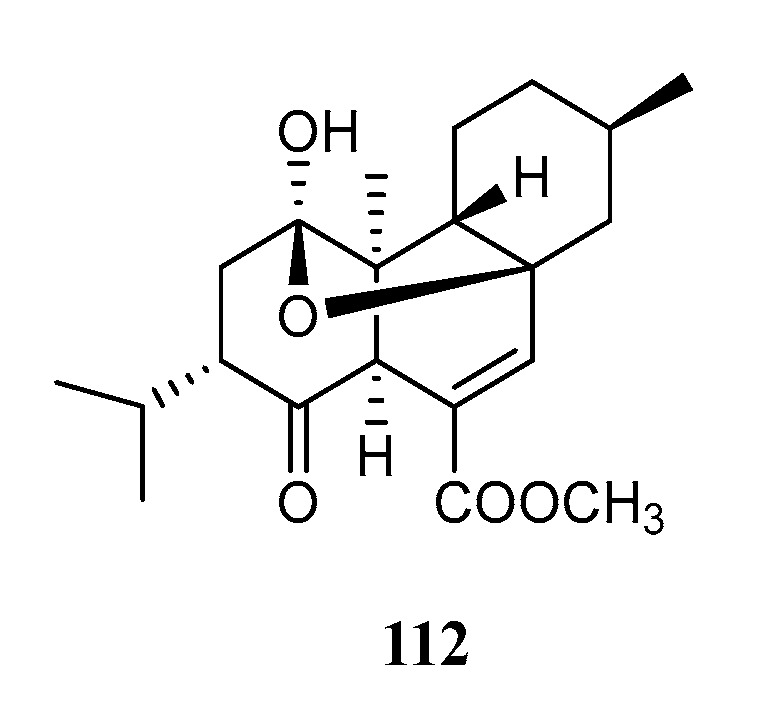
Structure of compound **112**, perhydrophenanthrane diterpenoid from *S. trocheliophorum*.

Perhydrophenanthrane diterpenoid was extremely rare. So far, only one member (Figure 11), sarcophytin (**112**), was found in the soft coral *S. trocheliophorum*, which was collected off the coast of Madepam, Indian Ocean [15]. This compound was presumed to be the precursor of trocheliophorin (**1**). Unfortunately, no bioassay was performed on this diterpenoid.

### 4.3. Capnosane Diterpenoids

**Figure 12:**
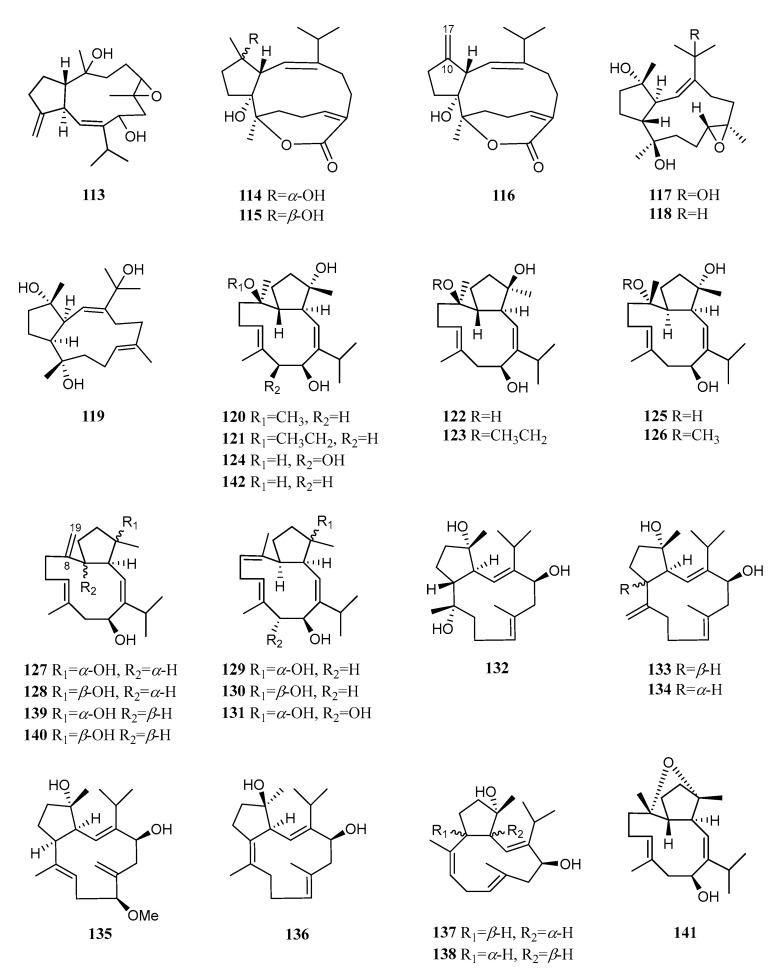
Structures of compounds **113**–**142**, capnosane diterpenoids from *S. trocheliophorum*.

The basic skeleton of capnosane, a 5/11-fused bicyclic system, has been considered as a C-3/C-7 cyclization derivative of cembrane. Up until now, twenty-eight capnosane diterpenoids have been reported in this species (Figure 12).

Compound **113** was the first capnosane diterpene found in the soft coral *S. trocheliophorum*, which was collected from the Andaman and Nicobar Islands, Indian Ocean [48]. Only the stereochemistry at the ring junction, together with the *E* conformation of the trisubstituted double bond, were established. A chemical investigation of a South China Sea sample from Yalong Bay, Hainan Island, yielded two new capnosane diterpenoids, sarsolilides B (**114**) and C (**115**), and one known analog, sarsolilide A (**116**) [5]. The absolute configuration of **114** was determined using the TDDFT ECD calculation, which was further confirmed using a single crystal X-ray diffraction experiment with Cu K*α* radiation [49]. Diterpenoids **114** and **116** showed PTP1B inhibitory activity, with IC_50_ values of 27.1 and 6.8 μM, respectively. The exomethylene Δ^10(17)^ of the capnosane framework might play a crucial role in the PTP1B inhibitory activity considering the decreased activity of compounds **114** and **115** with respect to that of **116**. Of more interest was the recognition of the stereochemistry of the hydroxyl group at C-10 by PTP1B. A compound with 10*S* configuration (in **114**) was preferable to that with 10*R* configuration (in **115**). A study on another Hainan specimen led to the discovery of three new capnosane type diterpenes: sarcophytrols A–C (**117**–**119**) [50]. Among them, **117** and **118** had an unusual 1*Z*-configuration double bond. Moreover, the absolute configuration of **117** was determined using X-ray diffraction analysis. In contrast to sarsolilide B (**114**), they were all inactive against the PTP1B enzyme. Regarding the structure–activity relationship of sarcophytrols, the lactone moiety of sarsolilide B (**114**) might be a key functional group.

Nineteen new capnosane cembranoids named trocheliophols A–S (**120**–**138**) together with sarcophytol L (**139**), 4-*epi*-sarcophytol L (**140**), and sarcophyolides B (**141**) and C (**142**) were isolated from the South China Sea soft coral *S. trocheliophorum* collected off the coast of Weizhou Island [6]. The absolute configurations of compounds **120**, **121**, **124**, **125**, **131**, **132**, **134**, and **138** were determined using X-ray diffraction analysis, Snatzke’s method, and the modified Mosher method, respectively. In the bioassays for the inhibitory effects against inflammation-related NF-κB, metabolites **124**, **125,** and **132** showed weak inhibitory rates of 11%, 29%, and 14% at 10 μM. Moreover, compounds **127**, **128**, **131**, **133**, **134,** and **137**–**140** exhibited antibacterial effects against *Xanthomonas vesicatoria*, *Agrobacterium tumefaciens*, *Pseudomonas lachrymans*, *B. subtilis*, and *S. aureus*, with MIC values ranging from 8 to 32 μg/mL. A preliminary analysis of the structure–activity relationships revealed that the exomethylene Δ^8(19)^ of the capnosane skeleton enhanced the antibacterial activity. Moreover, compound **138**, the only one with H-3*β* orientation, displayed the most potent inhibitory activity against the selected bacteria.

### 4.4. Sarsolenane Diterpenoids

**Figure 13:**
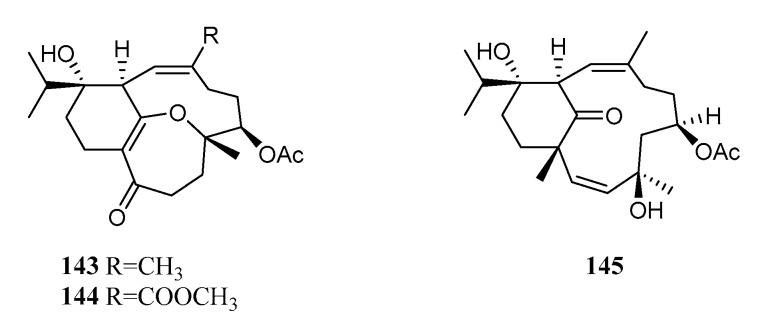
Structures of compounds **143**–**145**, sarsolenane diterpenoids from *S. trocheliophorum*.

To the best of our knowledge, only three sarsolenane diterpenoids [dihydrosarsolenone (**143**), methyl dihydrosarsolenoneate (**144**) and secodihydrosarsolenone (**145**), Figure 13 were isolated from a Chinese *S. trocheliophorum* sample collected in Yalong Bay, Hainan Island, South China Sea [5,51]. The assignment of absolute configuration of **143** was accomplished using the TDDFT ECD calculation. All of the metabolites were evaluated for inhibitory activity against PTP1B. Compound **145** exhibited PTP1B inhibitory activity with the IC_50_ value of 13.7 μM, whereas the other two were inactive (IC_50_ ≥ 50 μM). The ring cleavage of the *α*,*β*-unsaturated-*β*-ether ketone moiety in sarsolenane diterpenes might increase the PTP1B inhibitory activity due to the observation of considerable activity for **145** and no activity for **143** and **144**. A computational calculation gave an insight into the binding mode. It suggested a crucial role of the residues Tyr46, Arg221, and Ser216 in ligand–receptor binding to fulfill the inhibitory activity of the metabolite **145** towards PTP1B.

### 4.5. Sarcotroane Diterpenoids

**Figure 14:**
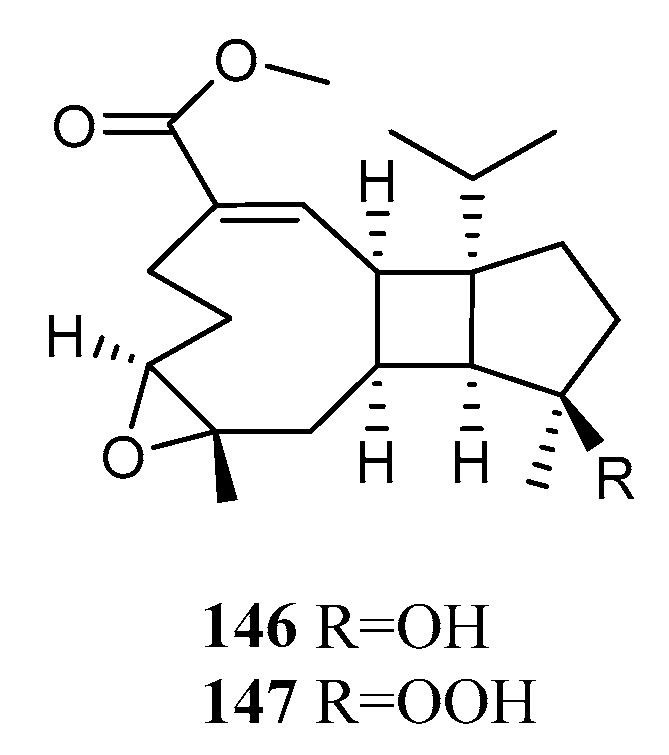
Structures of compounds **146** and **147**, sarcotroane diterpenoids from *S. trocheliophorum*.

Two unprecedented diterpenoids, methyl sarcotroates A (**146**) and B (**147**) (Figure 14), possessed a tetradecahydrocyclopenta[3′,4′]cyclobuta[1′,2′:4,5]cyclonona[1,2-b] oxirene ring system, which was named a sarcotroane. Both were given by the Hainan soft coral *S. trocheliophorum*. The TDDFT ECD calculation approach was used to determine the absolute configuration of compound **146**. Diterpenoid **147** exhibited significant inhibitory activity against PTP1B (IC_50_ = 6.97 μM), being similar to that of the positive control oleanolic acid [4]. This result indicated that the hydroperoxide group in the sarcotroane skeleton could enhance the PTP1B inhibitory activity.

## 5. Biscembranoids

**Figure 15:**
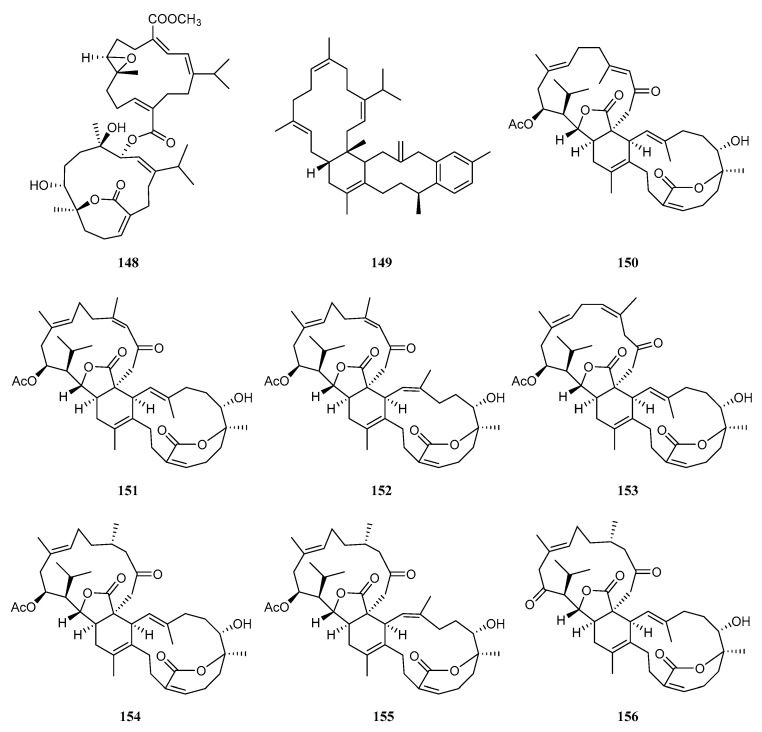
Structures of compounds **148**–**156**, biscembranoids from *S. trocheliophorum*.

Nine biscembranoids have been reported from the octocroal *S. trocheliophorum* (Figure 15). According to the different dimerization patterns, these biscembranoids could be divided into two types. The first type of cembrane dimers was formed by two cembranoid units connected through an ester linkage. The member of this category was bissartrolide (**148**), which was isolated from a South China Sea sample collected in Yalong Bay, Hainan Island [47]. Compound **148** was evaluated for antibacterial activity against the methicillin-sensitive *S. aureus* Newman strain, but it was inactive.

The second type of biscembranoid was dimerized via the Diels–Alder reaction with two monomers. Due to the structural diversity of the monomeric terpenes, this subgroup was further categorized by two different subclasses. The first carbon framework was trocheliane, which is named as it is merely one representative trocheliane (**149**). This compound was isolated from a Red Sea specimen and possessed an unprecedented tetracyclic hydrocarbon skeleton. This skeleton was proposed to be the Diels–Alder addition adduct of two cembrene-C (**16**) isomers accompanied with rearrangement and aromatization [37]. This hydrocarbon dimer showed promising activity against two multidrug resistant bacteria: *A. baumannii* and *S. aureus* (the diameters of inhibition zones were 18 ± 3.2, 18 ± 1.4 mm, respectively). The second framework was named glaucumane, the first two members of which were glaucumolides A (**150**) and B (**151**) from the soft coral *Sarcophyton glaucum* [52]. The proposed biosynthesis pathway of glaucumane biscembranoids was unique, which involved a *ε*-lactone cembrane as a diene monomer and a *γ*-lactone cembrane as a dienophile monomer. During the chemical investigation of a South China Sea soft coral *S. trocheliophorum* collected on the Xisha Islands, glaucumolides A (**150**) and B (**151**) and five new ones, bistrochelides A–E (**152**−**156**), were encountered [7]. Their absolute configurations were determined using X-ray crystal diffraction and TDDFT/ECD calculations. In in vitro immunomodulatory screening, compounds **150** and **154** significantly induced the proliferation of CD3^+^ T cells, while compound **152** significantly increased the CD4^+^/CD8^+^ ratio at 3.0 μM.

## 6. Conclusions

The abundant production and accumulation of terpenoids reported from the soft coral *S. trocheliophorum* is remarkable and intriguing. At present, 156 terpenoids have been encountered in the soft coral *S. trocheliophorum*, which indicates the productivity of this species. As shown in Table S1, the most typical terpenoids are macrocyclic cembrane diterpenes. Although other types of terpenoids are rarely encountered, they display a variety of unique carbon frameworks. Due to their novel structures, terpenoids from *S. trocheliophorum,* such as sarcophytin [53], are attractive targets for synthetic chemists.

It is interesting to notice that the chemical profiles of the title soft corals from different waters are different. For example, two *S. trocheliophorum* samples which grew in adjacent areas of the Red Sea (Jeddah, Saudi Arabia [16] and Hurgada, Egypt [17]) were afforded different chemotypes of sesquiterpenoids (Table S1). Moreover, the South China Sea *S. trocheliophorum* specimen yielded capnosane, sarsolenane, and sarcotroane diterpenoids [4,5] besides the common cembranoids [26], which were completely distinctive compared to that of the above-mentioned Red Sea sample [16]. In addition, the structural types of cembranoids from these two specimens were different. As reported, the *ε*-lactone and other miscellaneous cembranoids only existed in the South China Sea sample [25,26,29,47]. As shown in Figure 15, it was also obvious to find the influence of the geographical location on the types of biscembranoids. Two South China Sea specimens possessed bissartrane- [47] and glaucumane-type [7] biscembranoids, whereas the Red Sea specimen had a trocheliane-type biscembranoid. The impact of geographical location on the biscembranoids was also observed in the two South China Sea samples. The soft coral from Hainan Island afforded the bissartrane biscembranoid [47], while the one from the Xisha Islands yielded the glaucumane biscembranoids [7]. This probably reflects the existence of different metabolic processes in different inhabiting environments. Moreover, the impacts of temporal variations on the secondary metabolite production were also found. For instance, two South China Sea samples collected in Yalong Bay, Hainan Island, in different seasons (February [4], May [24], respectively) of the same year (Table S1), yielded diterpenes with completely distinct skeletons. Recently, Satheesh and Ba-Akdah also disclosed that the temporal variations significantly influenced the antifouling activity of the crude extracts of the soft coral *S. trocheliophorum* collected off the Jeddah coast of Saudi Arabia [54].

The terpenoids from *S. trocheliophorum* were screened for a vast array of potential pharmacological activities including PTP1B inhibitory, neuroprotective, cytotoxic, anti-inflammatory, antibacterial, antivirus, and immunomodulatory activities. As recorded in the literature, the majority of the reported compounds were inactive. These findings indicate that further substantial efforts are necessary to explore their unknown physiological roles.

Metabolites such as sarcophine (**77**) [16,35] and trocheliane (**149**) [37] exhibited significant biological activities, which could be developed as new drug leads. However, due to the low yields, a fairly large quantity of the coral organisms would be in demand. One way to accumulate the desired materials is to conduct heterologous expression of terpene biosynthetic genes for secondary metabolite production. Recently, Schmidt and co-workers found terpene synthase (TPS) genes in the genomes and transcriptomes of the soft coral *S. trocheliophorum*. In addition, the coral terpene cembrene-C (**16**) was successfully synthesized by incubating geranylgeranyl pyrophosphate (GGPP) with the purified coral enzyme EcTPS6 [55]. This work inspired us to explore more terpene biosynthetic gene clusters of this species, which might be in immense demand apart from the chemo- and bio-investigations.

## Data Availability

Not applicable.

## Supplementary Materials

The following supporting information can be downloaded at https://www.mdpi.com/article/10.3390/md21010030/s1: Table S1: Terpenoidal metabolites isolated from the soft coral *Sarcophyton trocheliophorum*.
