# Supplementary material for: The Soft Coral Sarcophyton trocheliophorum: A Warehouse of Terpenoids with Structural and Pharmacological Diversity
Source: Mar Drugs. 2022 Dec 29;21(1):30. doi: 10.3390/md21010030 (PMC9865811; doi:10.3390/md21010030)
Supplement: Supplementary file 1 [file marinedrugs-21-00030-s001.zip › marinedrugs-2095228-supplementary.pdf]

Supplementary Materials

Table S1. Terpenoidal metabolites isolated from the soft coral *Sarcophyton trocheliophorum*.

No.	Name	Locality	Bioassay	Class	Ref.
**1**	trocheliophorin	Kurside Island, India	-	trocheliophorane sesquiterpenoid	[15]
**2**	palustrol	Jeddah, Saudi Arabia	antibacterial, antifungal, antitumor, antifeedant	aromadendrane sesquiterpenoid	[16]
**3**	alloaromadendrene	Hurgada, Egypt	-	aromadendrane sesquiterpenoid	[17]
**4**	*β*-elemene	Hurgada, Egypt	-	elemane sesquiterpenoid	[17]
**5**	*trans*-caryophyllene	Hurgada, Egypt	-	caryophillane sesquiterpenoid	[17]
**6**	bisabolene	Hurgada, Egypt	-	bisabolane sesquiterpenoid	[17]
**7**	trocheliophorol	Seychelles Islands, Indian Ocean	-	cembrane diterpenoid	[3] [18]
		Orpheus Island, Australia	antifeedant	cembrane diterpenoid	[20]
**8**	thunbergol	Orpheus Island, Australia	antifeedant		[20]
**9**	7,8-epoxy-1*E*,3*E*,11*E*-cembratriene-15-ol	Orpheus Island, Australia	antifeedant	cembrane diterpenoid	[20]
		Pulau Hantu, Singapore	cytotoxic		[8]
**10**	13*S*-hydroxy-(-)-neocembrene	Seychelles Islands, Indian Ocean	cytotoxic	cembrane diterpenoid	[21]
**11**	13*S*-hydroxy-11,12-epoxy-(-)-neocembrene	Seychelles Islands, Indian Ocean	cytotoxic	cembrane diterpenoid	[21] [22]
**12**	neocembrene	Seychelles Islands, Indian Ocean	cytotoxic	cembrane diterpenoid	[21] [22]
**13**	(7*R*,8*R*,14*S*,1*E*,3*E*,11*E*)-7,8-epoxycembra-l,3,11-trien-14-ol	Orpheus Island, Australia	cytotoxic	cembrane diterpenoid	[23]
**14**	(7*R*,8*R*,l4*S*,l*E*,3*E*,11*E*)-14-acetoxy-7,8-epoxycembra-l,3,11-triene	Orpheus Island, Australia	cytotoxic	cembrane diterpenoid	[23]
**15**	(7*R*,14*S*,1*E*,3*E*,8*E*,11*E*)-cembra-1,3,8,11-tetraene-7,14-diol	Orpheus Island, Australia	cytotoxic	cembrane diterpenoid	[23]
**16**	isoneocembrene/cembrene-C	Pulau Hantu, Singapore	cytotoxic	cembrane diterpenoid	[8]
		Hainan Island, South China Sea	PTP1B inhibitory		[25]
		Jeddah, Saudi Arabia	antibacterial, antifungal, antifeedant, cytotoxic		[16]
**17**	7,8-epoxy-1*E*,3*E*,11*E*-cembratriene	Pulau Hantu, Singapore	cytotoxic	cembrane diterpenoid	[8]

No.	Name	Locality	Bioassay	Class	Ref.
**18**	yalongene A	Hainan Island, South China Sea	neuroprotective	cembrane diterpenoid	[24]
**19**	yalongene B	Hainan Island, South China Sea	neuroprotective	cembrane diterpenoid	[24]
**20**	sarcophytonolide M	Hainan Island, South China Sea	PTP1B inhibitory	cembrane diterpenoid	[4]
**21**	sarcophytonolide N	Hainan Island, South China Sea	PTP1B inhibitory, cytotoxic	cembrane diterpenoid	[25]
**22**	sarcophytonolide O	Hainan Island, South China Sea	PTP1B inhibitory, cytotoxic	cembrane diterpenoid	[25]
**23**	sarcophytonolide P	Hainan Island, South China Sea	PTP1B inhibitory, cytotoxic	cembrane diterpenoid	[25]
**24**	sarcophytonolide Q	Hainan Island, South China Sea	PTP1B inhibitory, cytotoxic	cembrane diterpenoid	[25]
**25**	sarcophytonolide S	Hainan Island, South China Sea	PTP1B inhibitory	cembrane diterpenoid	[26]
**26**	sarcophytonolide T	Hainan Island, South China Sea	PTP1B inhibitory	cembrane diterpenoid	[26]
**27**	sarcophytonolide U	Hainan Island, South China Sea	PTP1B inhibitory	cembrane diterpenoid	[26]
**28**	(*E*,*E*,*E*)-1-isopropenyl-4,8,12-trimethylcyclotetradeca-3,7,11-triene	Hainan Island, South China Sea	PTP1B inhibitory, cytotoxic	cembrane diterpenoid	[25]
**29**	sarcophytonolide A	Hainan Island, South China Sea	-	cembrane diterpenoid	[26]
**30**	(*E*,*E*,*E*)-7,8-epoxy-l-isopropyl-4,8,12-trimethylcyclotetradeca-l,3,11-triene	Hainan Island, South China Sea	-	cembrane diterpenoid	[26]
**31**	sarcophytrol D	Hainan Island, South China Sea	PTP1B inhibitory	cembrane diterpenoid	[27]
**32**	sarcophytrol E	Hainan Island, South China Sea	PTP1B inhibitory	cembrane diterpenoid	[27]
**33**	sarcophytrol F	Hainan Island, South China Sea	PTP1B inhibitory	cembrane diterpenoid	[27]
**34**	sarcophytrol G	Hainan Island, South China Sea	PTP1B inhibitory	cembrane diterpenoid	[28]
**35**	sarcophytrol H	Hainan Island, South China Sea	PTP1B inhibitory	cembrane diterpenoid	[28]
**36**	sarcophytrol I	Hainan Island, South China Sea	PTP1B inhibitory	cembrane diterpenoid	[28]
**37**	sarcophytrol J	Hainan Island, South China Sea<br>Ximao Island, South China Sea	PTP1B inhibitory<br>cytotoxic	cembrane diterpenoid	[28]<br>[31]
**38**	sarcophytrol K	Hainan Island, South China Sea	PTP1B inhibitory	cembrane diterpenoid	[28]
**39**	sarcophytrol L	Hainan Island, South China Sea	PTP1B inhibitory	cembrane diterpenoid	[28]
**40**	11,12-epoxy-1(*E*),3(*E*),7(*E*)-cembratrien-15-ol	Hainan Island, South China Sea	PTP1B inhibitory	cembrane diterpenoid	[27]
**41**	sinugibberol	Hainan Island, South China Sea	PTP1B inhibitory	cembrane diterpenoid	[27]
**42**	crassumol A	Hainan Island, South China Sea<br>Ximao Island, South China Sea	PTP1B inhibitory<br>cytotoxic	cembrane diterpenoid	[28]<br>[31]
**43**	sarglaucol	Hainan Island, South China Sea	PTP1B inhibitory	cembrane diterpenoid	[29]

No.	Name	Locality	Bioassay	Class	Ref.
**44**	7*R*[*],8*R*[*]-epoxy-isoneocembyance A-16-oic methyl ester	Hainan Island, South China Sea	cytotoxic, acetylcholinesterase inhibitory	cembrane diterpenoid	[30]
**45**	4-*epi*thunbergol	Hainan Island, South China Sea	cytotoxic, acetylcholinesterase inhibitory	cembrane diterpenoid	[30]
**46**	*cis*-cembrene C	Hurgada, Egypt	antifeedant, antibacterial, antifungal	cembrane diterpenoid	[17]
**47**	*cis*-cembrenene C	Hurgada, Egypt	antifeedant, antibacterial, antifungal	cembrane diterpenoid	[17]
**48**	(+)-sarcophytol A	Hurgada, Egypt	antifeedant, antibacterial, antifungal	cembrane diterpenoid	[31]
**49**	ximaosarcophytol A	Ximao Island, South China Sea	cytotoxic	cembrane diterpenoid	[32]
**50**	ximaosarcophytol B	Ximao Island, South China Sea	cytotoxic	cembrane diterpenoid	[32]
**51**	15-hydroxycembra-1,3,7,11-tetraene	Ximao Island, South China Sea	cytotoxic	cembrane diterpenoid	[32]
**52**	1,13-di-*epi*-13-acetoxy launine P	Xisha Islands, South China Sea	antibacterial, antiviral	cembrane diterpenoid	[33]
**53**	13-oxo-thunbergol	Xisha Islands, South China Sea	antibacterial, antiviral	cembrane diterpenoid	[33]
**54**	launine P	Xisha Islands, South China Sea	antibacterial, antiviral	cembrane diterpenoid	[33]
**55**	(+)-sarcophytoxide	Leti, Indonesia<br>Orpheus Island, Australia	-<br>-	cembrane diterpenoid	[3]<br>[23]
**56**	isosarcophytoxide	Leti, Indonesia<br>Kenting, Taiwan Island	-<br>cytotoxic	cembrane diterpenoid	[3]<br>[34]
**57**	16-deoxysarcophine	Kenting, Taiwan Island	cytotoxic	cembrane diterpenoid	[35]
**58**	(–)-sarcophytoxide	Hainan Island, South China Sea	cytotoxic	cembrane diterpenoid	[30]
**59**	sarcophytrol M	Hainan Island, South China Sea	cytotoxic, PTP1B inhibitory, antibacterial	cembrane diterpenoid	[29]
**60**	sarcophytrol N	Hainan Island, South China Sea	cytotoxic, PTP1B inhibitory, antibacterial	cembrane diterpenoid	[29]
**61**	sarcophytrol O	Hainan Island, South China Sea	cytotoxic, PTP1B inhibitory, antibacterial	cembrane diterpenoid	[29]
**62**	sarcophytrol P	Hainan Island, South China Sea	cytotoxic, PTP1B inhibitory, antibacterial	cembrane diterpenoid	[29]

No.	Name	Locality	Bioassay	Class	Ref.
**63**	isocrassumol B	Xisha Islands, South China Sea	antibacterial, antiviral	cembrane diterpenoid	[33]
**64**	sarcotrocheliol acetate	Jeddah, Saudi Arabia	antibacterial, antifeedant, cytotoxic	cembrane diterpenoid	[16]
		Hurghada, Egypt	antibacterial		[31] [38]
**65**	sarcotrocheliol	Jeddah, Saudi Arabia	antibacterial, antifeedant, cytotoxic	cembrane diterpenoid	[16]
		Hurghada, Egypt	cytotoxic, anti-leishmanial		[36] [39]
**66**	sarcotrocheldiol A	Jeddah, Saudi Arabia	antibacterial	cembrane diterpenoid	[37]
**67**	sarcotrocheldiol B	Jeddah, Saudi Arabia	antibacterial	cembrane diterpenoid	[37]
**68**	9-hydroxy-10,11-dehydro-sarcotrocheliol	Hurghada, Egypt	antibacterial	cembrane diterpenoid	[31]
**69**	9-hydroxy-7,8-dehydro-sarcotrocheliol	Hurghada, Egypt	antibacterial	cembrane diterpenoid	[36] [38]
**70**	8,9-expoy-sarcotrocheliol acetate	Hurghada, Egypt	antibacterial	cembrane diterpenoid	[38]
**71**	sarcopyranoid A	Hurghada, Egypt	cytotoxic, antiviral	cembrane diterpenoid	[39]
**72**	sarcophytrol Q	Hainan Island, South China Sea	cytotoxic, PTP1B inhibitory, antiviral	cembrane diterpenoid	[29]
**73**	sarcophytrol R	Hainan Island, South China Sea	cytotoxic, PTP1B inhibitory, antiviral	cembrane diterpenoid	[29]
**74**	sarcophytrol S	Hainan Island, South China Sea	cytotoxic, PTP1B inhibitory, antiviral	cembrane diterpenoid	[29]
**75**	(+)-isosarcophine	Kenting, Taiwan Island	cytotoxic	cembrane diterpenoid	[34]
**76**	7*β*,8*α*-dihydroxydeepoxysarcophine	Kenting, Taiwan Island	cytotoxic	cembrane diterpenoid	[35]
**77**	sarcophine	Kenting, Taiwan Island	cytotoxic	cembrane diterpenoid	[35]
		Pulau Hantu, Singapore	cytotoxic		[8]
		Jeddah, Saudi Arabia	antibacterial, antifungal, cytotoxic, antifeedant		[16]
**78**	(-)-sartrochine	South China Sea	cytotoxic, antibacterial	cembrane diterpenoid	[40]
**79**	sarcophytonolide J	Hainan Island, South China Sea	PTP1B inhibitory, cytotoxic	cembrane diterpenoid	[25]
**80**	sarcophytonolide R	Hainan Island, South China Sea	cytotoxic, PTP1B inhibitory	cembrane diterpenoid	[25]
**81**	*ent*-sarcophine	Hainan Island, South China Sea	acetylcholinesterase inhibitory, cytotoxic	cembrane diterpenoid	[30]

No.	Name	Locality	Bioassay	Class	Ref.
**82**	2-hydroperoxysarcophine	Hainan Island, South China Sea	acetylcholinesterase inhibitory, cytotoxic	cembrane diterpenoid	[30]
**83**	trocheliol	Pingtung, Taiwan Island	-	cembrane diterpenoid	[41]
**84**	trocheliolide A	Lanyu Island, Taiwan	cytotoxic	cembrane diterpenoid	[43]
**85**	trocheliolide B	Lanyu Island, Taiwan	-	cembrane diterpenoid	[44]
**86**	7β-hydroxy-8α-methoxydeepoxysarcophine	Gulf of Suez, Egypt	-	cembrane diterpenoid	[45]
**87**	trochelioid A	Hurghada, Egypt	-	cembrane diterpenoid	[46]
**88**	trochelioid B	Hurghada, Egypt	-	cembrane diterpenoid	[46]
**89**	16-oxosarcophytonin E	Hurghada, Egypt	-	cembrane diterpenoid	[46]
**90**	8-*epi*-sarcophinone	Hurghada, Egypt	-	cembrane diterpenoid	[46]
**91**	7α,8α-sarcophine	Xisha Islands, South China Sea	antibacterial, antiviral	cembrane diterpenoid	[33]
**92**	sarcophytonin B	Xisha Islands, South China Sea	antibacterial, antiviral	cembrane diterpenoid	[33]
**93**	sartrolide A	Hainan Island, South China Sea	PTP1B inhibitory, cytotoxic, antibacterial	cembrane diterpenoid	[26]
**94**	sartrolide B	Hainan Island, South China Sea	PTP1B inhibitory, cytotoxic, antibacterial	cembrane diterpenoid	[26]
**95**	sartrolide C	Hainan Island, South China Sea	PTP1B inhibitory, cytotoxic, antibacterial	cembrane diterpenoid	[26]
**96**	sartrolide D	Hainan Island, South China Sea	PTP1B inhibitory, cytotoxic, antibacterial	cembrane diterpenoid	[26]
**97**	sartrolide E	Hainan Island, South China Sea	PTP1B inhibitory, cytotoxic, antibacterial	cembrane diterpenoid	[26]
**98**	sartrolide F	Hainan Island, South China Sea	PTP1B inhibitory, cytotoxic, antibacterial	cembrane diterpenoid	[26]
**99**	sartrolide G	Hainan Island, South China Sea	PTP1B inhibitory, cytotoxic, antibacterial	cembrane diterpenoid	[26]
**100**	sartrolide H	Hainan Island, South China Sea	PTP1B inhibitory, cytotoxic, antibacterial	cembrane diterpenoid	[47]
**101**	sartrolide I	Hainan Island, South China Sea	PTP1B inhibitory, cytotoxic, antibacterial	cembrane diterpenoid	[47]

No.	Name	Locality	Bioassay	Class	Ref.
**102**	sartrolide J	Hainan Island, South China Sea	PTP1B inhibitory, cytotoxic, antibacterial	cembrane diterpenoid	[47]
**103**	ketoemblide	Hainan Island, South China Sea	PTP1B inhibitory, cytotoxic, antibacterial	cembrane diterpenoid	[25]
**104**	4*Z*,12*Z*,14*E*-sarcophytolide	Hainan Island, South China Sea	PTP1B inhibitory, cytotoxic, antibacterial	cembrane diterpenoid	[25] [47]
**105**	sarcrassin D	Hainan Island, South China Sea	PTP1B inhibitory, cytotoxic, antibacterial	cembrane diterpenoid	[26] [47]
**106**	sarcrassin E	Hainan Island, South China Sea	PTP1B inhibitory, cytotoxic, antibacterial	cembrane diterpenoid	[25]
**107**	emblide	Hainan Island, South China Sea	PTP1B inhibitory, cytotoxic, antibacterial	cembrane diterpenoid	[26] [47]
**108**	sarcophytolide	Hainan Island, South China Sea	antibacterial	cembrane diterpenoid	[26]
**109**	deacetylemblide	Hainan Island, South China Sea	PTP1B inhibitory, cytotoxic, antibacterial	cembrane diterpenoid	[47]
**110**	sarcophytrol T	Hainan Island, South China Sea	PTP1B inhibitory, cytotoxic, antibacterial	cembrane diterpenoid	[29]
**111**	sarcophytrol U	Hainan Island, South China Sea	PTP1B inhibitory, cytotoxic, antibacterial	cembrane diterpenoid	[29]
**112**	sarcophytin	Kurside Island, India	-	perhydrophenanthrane diterpenoids	[15]
**113**	unnamed	Andaman and Nicobar Islands, Indian Ocean	-	capnosane diterpenoids	[48]
**114**	sarsolilide B	Hainan Island, South China Sea	PTP1B inhibitory	capnosane diterpenoids	[5]
**115**	sarsolilide C	Hainan Island, South China Sea	PTP1B inhibitory	capnosane diterpenoids	[5]
**116**	sarsolilide A	Hainan Island, South China Sea	PTP1B inhibitory	capnosane diterpenoids	[5]
**117**	sarcophytrol A	Hainan Island, South China Sea	PTP1B inhibitory	capnosane diterpenoids	[50]
**118**	sarcophytrol B	Hainan Island, South China Sea	PTP1B inhibitory	capnosane diterpenoids	[50]
**119**	sarcophytrol C	Hainan Island, South China Sea	PTP1B inhibitory	capnosane diterpenoids	[50]
**120**	trocheliophol A	Weizhou Island, South China Sea	anti-inflammatory, antibacterial	capnosane diterpenoids	[6]
**121**	trocheliophol B	Weizhou Island, South China Sea	anti-inflammatory, antibacterial	capnosane diterpenoids	[6]

No.	Name	Locality	Bioassay	Class	Ref.
**122**	trocheliophol C	Weizhou Island, South China Sea	anti-inflammatory, antibacterial	capnosane diterpenoids	[6]
**123**	trocheliophol D	Weizhou Island, South China Sea	anti-inflammatory, antibacterial	capnosane diterpenoids	[6]
**124**	trocheliophol E	Weizhou Island, South China Sea	anti-inflammatory, antibacterial	capnosane diterpenoids	[6]
**125**	trocheliophol F	Weizhou Island, South China Sea	anti-inflammatory, antibacterial	capnosane diterpenoids	[6]
**126**	trocheliophol G	Weizhou Island, South China Sea	anti-inflammatory, antibacterial	capnosane diterpenoids	[6]
**127**	trocheliophol H	Weizhou Island, South China Sea	anti-inflammatory, antibacterial	capnosane diterpenoids	[6]
**128**	trocheliophol I	Weizhou Island, South China Sea	anti-inflammatory, antibacterial	capnosane diterpenoids	[6]
**129**	trocheliophol J	Weizhou Island, South China Sea	anti-inflammatory, antibacterial	capnosane diterpenoids	[6]
**130**	trocheliophol K	Weizhou Island, South China Sea	anti-inflammatory, antibacterial	capnosane diterpenoids	[6]
**131**	trocheliophol L	Weizhou Island, South China Sea	anti-inflammatory, antibacterial	capnosane diterpenoids	[6]
**132**	trocheliophol M	Weizhou Island, South China Sea	anti-inflammatory, antibacterial	capnosane diterpenoids	[6]
**133**	trocheliophol N	Weizhou Island, South China Sea	anti-inflammatory, antibacterial	capnosane diterpenoids	[6]
**134**	trocheliophol O	Weizhou Island, South China Sea	anti-inflammatory, antibacterial	capnosane diterpenoids	[6]
**135**	trocheliophol P	Weizhou Island, South China Sea	anti-inflammatory, antibacterial	capnosane diterpenoids	[6]
**136**	trocheliophol Q	Weizhou Island, South China Sea	anti-inflammatory, antibacterial	capnosane diterpenoids	[6]
**137**	trocheliophol R	Weizhou Island, South China Sea	anti-inflammatory, antibacterial	capnosane diterpenoids	[6]
**138**	trocheliophol S	Weizhou Island, South China Sea	anti-inflammatory, antibacterial	capnosane diterpenoids	[6]
**139**	sarcophytol L	Weizhou Island, South China Sea	anti-inflammatory, antibacterial	capnosane diterpenoids	[6]
**140**	4-*epi*-sarcophytol L	Weizhou Island, South China Sea	anti-inflammatory, antibacterial	capnosane diterpenoids	[6]
**141**	sarcophyolide B	Weizhou Island, South China Sea	anti-inflammatory, antibacterial	capnosane diterpenoids	[6]
**142**	sarcophyolide C	Weizhou Island, South China Sea	anti-inflammatory, antibacterial	capnosane diterpenoids	[6]
**143**	dihydrosarsolenone	Hainan Island, South China Sea	PTP1B inhibitory	sarsolenane diterpenoids	[5]
**144**	methyl dihydrosarsolenoneate	Hainan Island, South China Sea	PTP1B inhibitory	sarsolenane diterpenoids	[5]
**145**	secodihydrosarsolenone	Hainan Island, South China Sea	PTP1B inhibitory	sarsolenane diterpenoids	[51]
**146**	methyl sarcotroate A	Hainan Island, South China Sea	PTP1B inhibitory	sarcotroane diterpenoids	[4]
**147**	methyl sarcotroate B	Hainan Island, South China Sea	PTP1B inhibitory	sarcotroane diterpenoids	[4]
**148**	bissartrolide	Hainan Island, South China Sea	antibacterial	bissartrane biscembranoids	[47]
**149**	trocheliane	Jeddah, Saudi Arabia	antibacterial	trocheliane biscembranoids	[37]
**150**	glaucumolide A	Xisha Islands, South China Sea	immunomodulatory	glaucumane biscembranoids	[7]
**151**	glaucumolide B	Xisha Islands, South China Sea	immunomodulatory	glaucumane biscembranoids	[7]

No.	Name	Locality	Bioassay	Class	Ref.
**152**	bistrochelide A	Xisha Islands, South China Sea	immunomodulatory	glaucumane biscembranoids	[7]
**153**	bistrochelide B	Xisha Islands, South China Sea	immunomodulatory	glaucumane biscembranoids	[7]
**154**	bistrochelide C	Xisha Islands, South China Sea	immunomodulatory	glaucumane biscembranoids	[7]
**155**	bistrochelide D	Xisha Islands, South China Sea	immunomodulatory	glaucumane biscembranoids	[7]
**156**	bistrochelide E	Xisha Islands, South China Sea	immunomodulatory	glaucumane biscembranoids	[7]

* Note: In the table, '-' indicates no bioassay was performed.
